# Giant Cell Tumor of Lumbar Vertebrae on MR and 18F- FDG PET/CT: A Case Report and Literature Review

**DOI:** 10.5334/jbsr.3012

**Published:** 2023-03-06

**Authors:** Xiangxiang Liu, Cen Lou, Zhongke Huang

**Affiliations:** 1 Sir Run Run Shaw Hospital, Zhejiang University School of Medicine, CN

**Keywords:** giant cell tumor, vertebral tumors, PET/CT, MRI, benign fibrous histiocytoma

## Abstract

**Teaching Point:** Giant cell tumor of bone may show a moderate to high FDG uptake, and attention should be paid to differentiate from malignant tumors.

## Introduction

Giant cell tumor (GCT) is a common benign bone tumor, accounting for 5% of all primary bone tumors [1,2]. Vertebral giant cell tumor is a very rare tumor, and imaging studies are challenging because it shares features with some other spinal lesions. There are not many reports on giant cell tumor of the lumbar spine, and even fewer reports on PET/CT of spinal GCT [3,4,5,6]. Herein, we present a case of GCT of lumbar vertebrae on magnetic resonance imaging (MRI) and PET/CT, as well as review the literature.

## Case History

**Figure 1:**
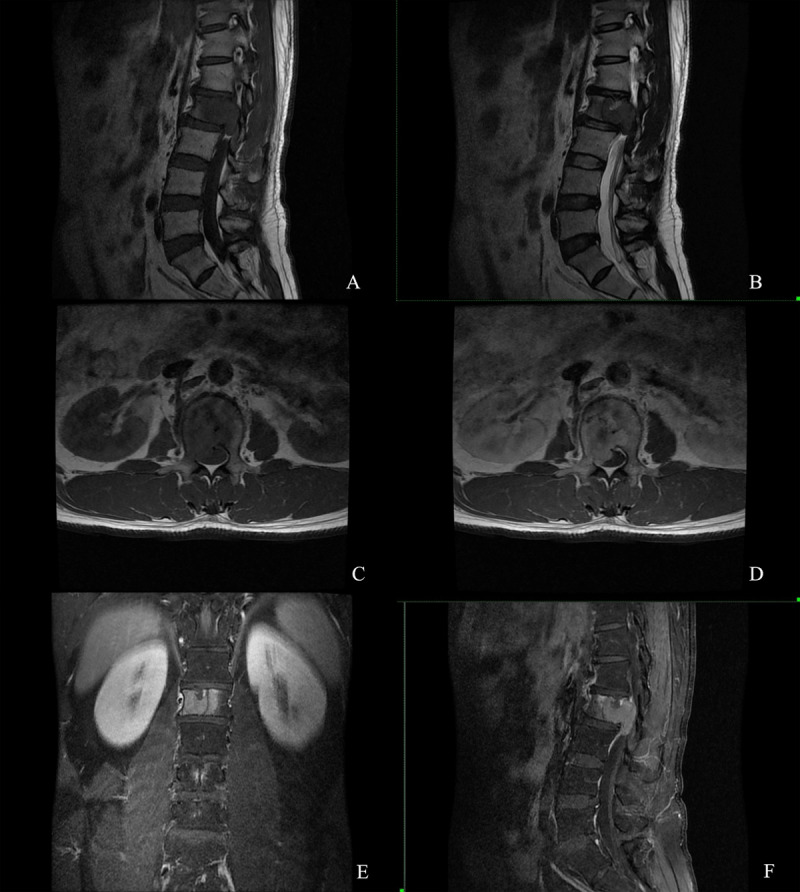
MRI.

**Figure 2:**
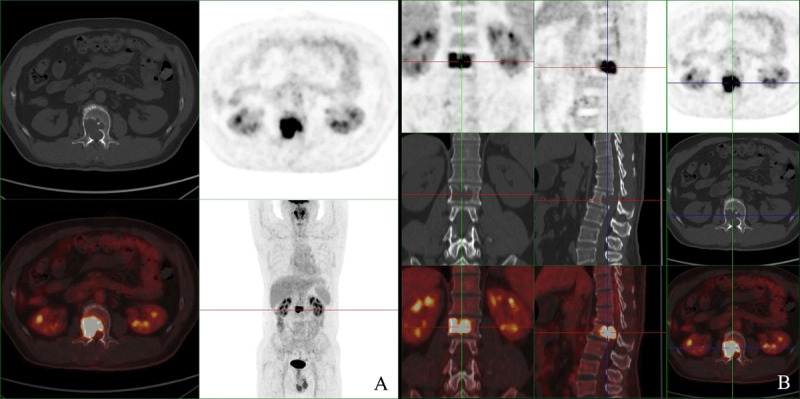
PET/CT.

A 45-year-old man with pain and numbness in the right lower limb for more than one year was referred for magnetic resonance imaging (MRI). MRI showed a large expansile lesion of the second lumbar vertebra (Figure 1, A sagittal T1; B sagittal T2, C axial T1 FSE). The right pedicle was involved, and part of the mass protruded into the spinal canal. The lesion was uniformly enhancing on contrast-enhanced MRI images (Figure 1 D–F). ^18^F-FDG PET/CT scan showed the expansile bone destruction of the second lumbar vertebra with increased FDG uptake (SUVmax: 11.8) (Figure 2). The cortex was eroded, with a residual bone crest.

**Figure 3:**
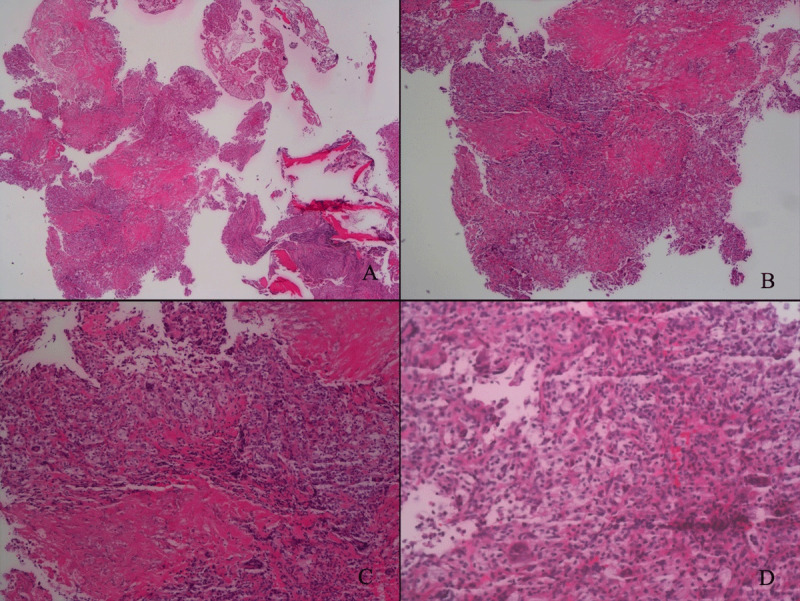
Pathological result.

The patient underwent vertebral resection. Pathological diagnosis was a benign fibrous histiocytoma (BFH) (now considered GCT) [7,8]. Light microscopy showed that spindle cells were arranged in a swirled structure (Figure 3). Osteoclast-type giant cells and foamy cells were scattered in the stroma.

## Comments

BFH shares common clinical symptoms, location, radiological characteristics, and histological features with GCT [8,9], causing difficulties in diagnosis. According to the latest WHO Classification of Tumors of Bone (2020), BFH of the spine is now classified as a heterogeneous GCT [10,11]. GCTs are rare benign tumors, but with aggressive behavior. Spinal GCTs are even rarer, reported in less than 3% of cases [12].

Despite benign in nature, GCTs generally exhibit high FDG uptake. A retrospective study of 20 patients from a single center showed that the SUVmax of spinal GCT ranged between 7.6 and 13.7, and the mean SUVmax was 10.4 ± 2.7 [4], consistent with our results (SUVmax: 11.8). Previous studies indicated that the high SUVmax of GCT was caused by overexpression of glucose transporter type 1 (GLUT-1) and hexokinase-2 in macrophages and giant cells, reactive fibroblast proliferation or enhanced angiogenesis [13]. High FDG uptake may cause a misdiagnosis of malignancy.

## Conclusion

GCT is a rare benign skeletal tumor. It shares features with some other malignant bone lesions on MRI and PET-CT and should be included in the differential diagnosis.

## References

[1] Lin P, Lin N, Teng W, et al. Recurrence of giant cell tumor of the spine after resection: A report of 10 cases. Orthop Surg. 2018; 10(2): 107–14. DOI: 10.1111/os.12375 29878714 PMC6001436

[2] Chakarun CJ, Forrester DM, Gottsegen CJ, Patel DB, White EA, Matcuk GR. Giant cell tumor of bone: Review, mimics, and new developments in treatment. Radiographics. 2013; 33(1): 197–211. DOI: 10.1148/rg.331125089 23322837

[3] Dejust S, Jallerat P, Soibinet-Oudot P, Jouannaud C, Morland D. Multimodality imaging features of a misleading sacral giant cell tumor in 18F-FDG PET/CT, bone scan, and MRI. Clin Nucl Med. 2020; 45(10): 800–1. DOI: 10.1097/RLU.0000000000003148 32604109

[4] Muheremu A, Ma Y, Huang Z, Shan H, Li Y, Niu X. Diagnosing giant cell tumor of the bone using positron emission tomography/computed tomography: A retrospective study of 20 patients from a single center. Oncol Lett. 2017; 14(2): 1985–8. DOI: 10.3892/ol.2017.6379 28781642 PMC5530223

[5] Hoshi M, Takada J, Oebisu N, Hata K, Ieguchi M, Nakamura H. Overexpression of hexokinase-2 in giant cell tumor of bone is associated with false positive in bone tumor on FDG-PET/CT. Arch Orthop Trauma Surg. 2012; 132(11): 1561–8. DOI: 10.1007/s00402-012-1588-2 22825642

[6] McKinney AM, Reichert P, Short J, et al. Metachronous, multicentric giant cell tumor of the sphenoid bone with histologic, CT, MR imaging, and positron-emission tomography/CT correlation. AJNR Am J Neuroradiol. 2006; 27(10): 2199–201. 17110693 PMC7977200

[7] Ezgu MC, Cicek AF, Yasar S. Benign fibrous histiocytoma of the cervical vertebra: A rare case. Neurol India. 2019; 67(1): 306–8. 30860146 10.4103/0028-3886.253644

[8] Mondal SK. Cytodiagnosis of benign fibrous histiocytoma of rib and diagnostic dilemma: A case report. Diagn Cytopathol. 2010; 38(6): 457–60. DOI: 10.1002/dc.21245 20014125

[9] Hattori T, Matsumine A, Uchida K, Nojima T, Sudo A. Benign fibrous histiocytoma of the talus: A case report. J Foot Ankle Surg. 2019; 58(4): 762–5. DOI: 10.1053/j.jfas.2018.11.009 30962105

[10] Ventura L, Petrella E, Piciucchi S, et al. Giant cell tumor of bone in an eighteenth-century Italian mummy. Virchows Arch. 2021; 479(6): 1255–61. DOI: 10.1007/s00428-021-03192-5 34462806 PMC8724190

[11] Choi JH, Ro JY. The 2020 WHO classification of tumors of soft tissue: Selected changes and new entities. Adv Anat Pathol. 2021; 28(1): 44–58. DOI: 10.1097/PAP.0000000000000284 32960834

[12] Montgomery C, Couch C, Emory CL, Nicholas R. Giant cell tumor of bone: Review of current literature, evaluation, and treatment options. J Knee Surg. 2019; 32(4): 331–6. DOI: 10.1055/s-0038-1675815 30449024

[13] Park HL, Yoo IR, Lee Y, Park SY, Jung CK. Giant cell tumor of the rib: Two cases of F-18 FDG PET/CT findings. Nucl Med Mol Imaging. 2017; 51(2): 182–5. DOI: 10.1007/s13139-016-0442-9 28559944 PMC5429297

